# Genome sequencing and annotation of a *Campylobacter coli* strain isolated from milk with multidrug resistance

**DOI:** 10.1016/j.gdata.2016.05.003

**Published:** 2016-05-05

**Authors:** Kun C. Liu, Karen C. Jinneman, Jason Neal-McKinney, Wen-Hsin Wu, Daniel H. Rice

**Affiliations:** Applied Technology Center, Pacific Regional Laboratory Northwest, US Food and Drug Administration, 22201 23rd Drive SE, Bothell, WA 98012, United States

**Keywords:** WGS (Whole Genome Sequencing), *C*. *coli* (*Campylobacter coli*), RAST (Rapid Annotation using Subsystem Technology)

## Abstract

As the most prevalent bacterial cause of human gastroenteritis, food-borne *Campylobacter* infections pose a serious threat to public health. Whole Genome Sequencing (WGS) is a tool providing quick and inexpensive approaches for analysis of food-borne pathogen epidemics. Here we report the WGS and annotation of a *Campylobacter coli* strain, FNW20G12, which was isolated from milk in the United States in 1997 and carries multidrug resistance. The draft genome of FNW20G12 (DDBJ/ENA/GenBank accession number LWIH00000000) contains 1, 855,435 bp (GC content 31.4%) with 1902 annotated coding regions, 48 RNAs and resistance to aminoglycoside, beta-lactams, tetracycline, as well as fluoroquinolones. There are very few genome reports of *C*. *coli* from dairy products with multidrug resistance. Here the draft genome of FNW20G12, a *C*. *coli* strain isolated from raw milk, is presented to aid in the epidemiology study of *C*. *coli* antimicrobial resistance and role in foodborne outbreak.

**Table:** 

Specifications	
Organism/cell line/tissue	Food-borne Bacterium pathogen
Sequencer or array type	Illumina MiSeq
Data format	Processed
Experimental factors	Bacterial strain
Experimental features	Assembled and annotated draft genome of a strain from milk with multidrug resistance
Consent	NA

**Accession number of deposited WGS genome**: DDBJ/ENA/GenBank under the accession LWIH00000000, version LWIH01000000.

## 1 Introduction

Campylobacteriosis is the primary cause of bacterial infections resulting in food-borne gastroenteritis with symptoms ranging from abdominal cramping, fever, to bloody diarrhea. In the United States, there are over 2.4 million people infected by *Campylobacter* each year [1]. Besides raw meat, primary risk factors of campylobacteriosis include consumption of raw milk, contaminated produce and water. Recently the role of *C*. *coli* in human disease has been increasingly recognized as it accounts for up to 25% of the total *Campylobacter*-associated diarrhea and has been reported to cause bacteremia, meningitis, spontaneous abortion and community acquired inflammatory enteritis [2], [3], [4]. Despite the application of Whole Genome Sequencing (WGS) on *Campylobacter* isolates, especially *C*. *jejuni* from clinical patients, genome reports of *C*. *coli* from dairy products with multidrug resistance are scarce. Here we announce the WGS and annotation of *C*. *coli* FNW20G12 strain, which was isolated from milk in the United States in 1997.

## 2 Experimental methods and results

The FNW20G12 strain was isolated and previously characterized according to the FDA Bacteriological Analytical Manual [5]. Prepared *Campylobacter* selective agar (catalog# B21727X, ThermoFisher Scientific Inc., Waltham, MA) and GasPak EZ Container System with Gas Sachets (Becton Dickinson Co., Franklin Lakes, NJ) were used to achieve microaerobic growth of FNW20G12. Genomic DNA was extracted with a QIAcube instrument (QIAGEN Inc., Valencia, CA) and quantified with a Qubit 3.0 fluorimeter (ThermoFisher Scientific Inc.). A library was then prepared with the Nextera XT sample preparation kit (Illumina, San Diego, CA) following manufacturer's instructions.

**Fig. 1:**
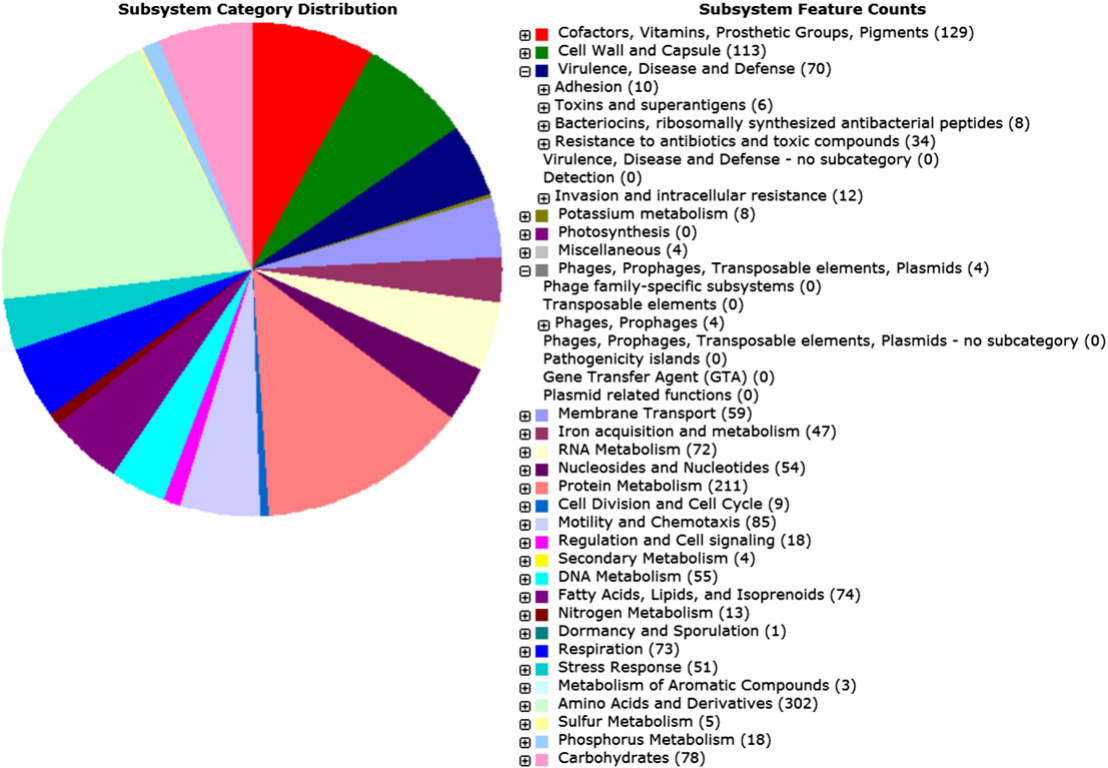
**Pie chart representing RAST subsystems in the *C***. ***coli* FNW20G12 genome**. The left pie chart shows the % distribution of 20 most abundant subsystems on the “category” level, each of which is represented by a particular color indicated in the right column showing the counts of features. A subsystem is a set of functional roles that together implement a specific biological process or structural complex, generally refereed as a pathway. The “Phages, Prophages, Transposable elements, Plasmids” category is expanded in the right column to show that there is no plasmid related functions annotated. The “Virulence, Disease and Defense” category is expended to show the virulence genes identified by RAST.

The genome of FNW20G12 was sequenced using a MiSeq instrument (Illumina) with 251 reading cycles. A total of 460,796,326 nucleotides were sequenced and 2,061,344 paired-end raw reads were produced with overall coverage of 312 ×. CLC Genomics Workbench v8.5.1 (QIAGEN Inc.) was employed to trim the index primers and a total of 2,059,260 filtered high quality reads were yielded with the median PHRED score greater than 30, showing the overall accuracy of base calling greater than 99.9%. There was no ambiguous base detected. *De novo* assembly was performed by CLC Genomics Workbench with word size of 45 and bubble sized of 98, and 327 contigs were obtained with a N_50_ contig length of 33,019 bp. We then removed contigs shorter than 200 bp, and removed a contig from human source identified by NCBI contamination screening pipeline. The final genome contained 1,855,435 bp in 324 contigs with GC content of 31.4% and the largest contig measured 157,691 bp. As shown in Fig. 1, the genome of FNW20G12 strain was annotated with a total of 312 subsystems, 1902 coding sequences (CDSs), and 48 RNAs with Rapid Annotation using Subsystem Technology (RAST) [6], [7], [8]. RAST also identified *C*. *coli* RM2228 as the closest neighbor of FNW20G12. Among the CDSs, there were conserved genes in metabolism and biosynthesis subsystems, as well as those involved in adhesion, virulence, and response to oxidation stress, such as *cad*F, *fla*C, and *sod*ABC. With RAST and ResFinder-2.1 [9], genes responsible for multidrug resistance, *aph*(3′)-III, *bla*OXA-61, *tet*O and *gyr*AB were identified in FNW20G12 with predicted resistance to aminoglycoside, beta-lactams, tetracycline and fluoroquinolones, respectively. Additionally, we found genes encoding efflux pumps and transporters (RND, MFS, DMT and MATE) which are associated with increased multidrug resistance. There was no plasmid replicon region identified in FNW20G12 genome using PlasmidFinder [10].

## 3 Accession number of nucleotide sequence

The draft genome assembled form Whole Genome Shotgun Sequencing of *C*. *coli* FNW20G12 strain has been deposited at DDBJ/ENA/GenBank under the accession LWIH00000000 with the version LWIH01000000.

## Conflict of interest

The authors declare that no conflict of interest exists about the work published in this paper.

## Disclaimer

The views presented in this work do not necessarily reflect those of the Food and Drug Administration, nor do the authors specifically endorse the listed instrumentation or products.
